# Numerate people are less likely to be biased by regular science reporting: the critical roles of scientific reasoning and causal misunderstanding

**DOI:** 10.1186/s41235-025-00641-6

**Published:** 2025-06-15

**Authors:** Olivia D. Perrin, Jinhyo Cho, Edward T. Cokely, Jinan N. Allan, Adam Feltz, Rocio Garcia-Retamero

**Affiliations:** 1 Department of Psychology, The University of Oklahoma, 455 West Lindsey Street, Dale Hall Tower, Norman, OK 73019 USA; 2 University of Southern California, Los Angeles, USA; 3 Clemson University, Clemson, USA; 4 University of Granada, Granada, Spain

**Keywords:** Numeracy, Risk literacy, Scientific reasoning, Causal theory errors

## Abstract

Numerate people tend to make more informed judgments and decisions because they are more risk literate (i.e., better able to evaluate and understand risk). Do numeracy skills also help people understand regular science reporting from mainstream news sources? To address this question, we investigated responses to regular science reports (e.g., excerpts from *CNN Health*), testing a cognitive model linking numeracy, scientific reasoning, judgment biases, and causal theory errors (i.e., interpreting correlational information as causal). In Study 1 (*n* = 200), structural equation modeling indicated that more numerate people were less likely to exhibit judgment biases because they were better at scientific reasoning, which helped them avoid causal misinterpretations. Study 2 (*n* = 342) cross-validated findings from Study 1, indicating that the link between numeracy and scientific reasoning was also associated with improved cognitive self-assessment (e.g., reduced overconfidence on comprehension judgments). Results indicate that more numerate people may generally be less likely to confuse correlation and causation in regular science reporting. Results also suggest that numerate people are more likely to have acquired scientific reasoning skills that more generally support risk literacy and knowledge acquisition, consistent with Skilled Decision Theory. Discussion focuses on implications for risk literacy research, and includes a Risk Literacy Difficulty Analysis indicating that more than half of the USA adult population may be likely to misunderstand common types of regular science reports.

**Supplementary Information:**

The online version contains supplementary material available at 10.1186/s41235-025-00641-6.

## Significance statement

Are some people more likely to misunderstand reputable science reporting, such as secondary reports about scientific findings distributed by mainstream news sources? In two studies, we measured cognitive skills (e.g., risk literacy as measured by the Berlin Numeracy Test) and investigated people’s susceptibility to potentially costly biases by asking young adults to review secondary science reports based on actual reporting (e.g., *CNN Health*). Consistent with previous research we found that many people confused correlational evidence with causal evidence (i.e., causal theory errors), which increased their susceptibility to a wide range of biases (e.g., judgment errors, overconfidence, misinformation propagation). However, we also found that risk literacy skills were consistently associated with scientific reasoning quality and reduced bias susceptibility. Results suggest that risk literacy skills may generally predict how likely it is that people will experience confusion and bias when reading secondary science reports. While misinformation and disinformation continue to threaten informed decision-making, the current findings indicate that even reputable science reporting may routinely bias a large proportion of adults (e.g., those who are not highly risk literate).

**Supplementary Information:**

The online version contains supplementary material available at 10.1186/s41235-025-00641-6.

## Introduction

Many people learn about scientific findings from secondary sources such as regular science reports—i.e., typical journalistic reports created and distributed by mainstream news and mass media providers (Funk et al., 2017; Hendriks et al., 2020; National Science Board, 2018; Pavelle & Wilkinson, 2020). However, research indicates people’s ability to accurately interpret regular science reporting varies as a function of individual differences in education, knowledge, skills, abilities, and cognitive styles (e.g., Bromme & Goldman, 2014; Miller, 1998; Stanovich & West, 1997). For example, specialized scientific reasoning skills appear to influence people’s ability to interpret and understand the quality of scientific evidence (Drummond & Fischhoff, 2017). As such, some people may be more likely to misunderstand some scientific information about personally relevant risks, which in turn could lead to costly judgment errors and biases (e.g., misinformed attitudes about risk mitigation).

Theoretically, statistical numeracy skills (i.e., practical probabilistic and inductive reasoning skills) may be another factor that could help people avoid misinterpretations of regular science reporting because numeracy is one of the most robust predictors of general decision making skill, including the ability to evaluate and understand risk (i.e., risk literacy; Cokely et al., 2012, 2018; see RiskLiteracy.org). For example, previous studies have shown that statistical numeracy skills are related to many high-stakes decisions, including quickly seeking medical care during a heart attack and accurately interpreting complex medical information (Peters, 2012, 2020; Petrova et al., 2016, 2017; Reyna et al., 2009). There are many cognitive mechanisms that may help explain the link between statistical numeracy and decision quality (e.g., Cokely & Kelley, 2009; Garcia-Retamero et al., 2019; Peters, 2020; Peters et al., 2006; Petrova et al., 2023; Reyna & Brainerd, 2023). Some accounts emphasize the influence of precise affective representations of numeric magnitudes (Peters et al., 2008) or the automatic influences of verbatim and gist processing in memory (i.e., Fuzzy Trace Theory; Reyna et al., 2009), while others have emphasized differences in people’s capacity for overriding emotions and intuitions in the service of logical reasoning (e.g., Dual Systems Theory; Stanovich & West, 2000). However, according to Skilled Decision Theory (Cokely et al., 2018; see also Cho et al., 2023), one’s deliberatively acquired *representative understanding* is by far the most influential factor that gives rise to skilled judgment and decision-making of experts and non-experts alike (e.g., Chase & Simon, 1973; Ericsson et al., 2006, 2007, 2018, Klein, 1999; but see also Chi et al., 1981, 1982; Cheng & Holyoak, 1985 for related accounts of knowledge representations, problem solving, and reasoning schemas). Put simply, because numerate people are more risk literate they tend to deliberate more on risk information, and can therefore develop a more accurate and intuitive understanding of most risk-relevant topics, which then helps them use heuristics to make informed judgments and decisions (Gigerenzer & Gaissmaier, 2011, Gigerenzer et al., 2000; see Cho et al., 2023 for an example in climate change and Petrova et al., 2017 for an example in medical decision-making).

Given that more numerate people are better able to independently acquire, evaluate, and understand risk information, one open question is whether numerate people might also benefit more from regular science reporting. Some evidence suggests this kind of relationship may be likely. Numerate people tend to correctly interpret many kinds of professionally developed risk and science communications (e.g., health, finance, and safety; Garcia-Retamero & Cokely, 2013, 2017; Garcia-Retamero et al., 2020; National Academies of Sciences, 2017; Peters, 2012, 2020; Reyna et al., 2009; see also Bruine de Bruin & Bostrom, 2013). Numerate people are less susceptible to biases related to how numerical information is presented (Garcia-Retamero & Cokely, 2011, 2014, 2015; Garcia-Retamero & Galesic, 2010; Peters & Bunquin, 2024; Petrova et al., 2015, 2016) and are less susceptible to misinformation and motivated reasoning effects associated with the use of some alternative or social media sources (Cho et al., 2023; Kahne & Bowyer, 2017; Roh et al., 2015; Roozenbeek et al., 2020; Taber & Lodge, 2006; Van der Linden, 2023). Numerate people also tend to be less susceptible to folk science myths and generally acquire more accurate knowledge about many diverse hazards and risks (Allan et al., 2017; Feltz, 2016; Feltz et al., 2022, 2023; Garrido et al., 2021; Ghazal et al., 2014; Grounds & Joslyn, 2018; Michal & Shah, 2024; Okan et al., 2012, 2016; Petrova et al., 2017, 2018; Ramasubramanian et al., 2019; Tanner & Feltz, 2022; Tanner et al., 2024, 2025). However, we have not been able to find any published research directly testing the relations among numeracy, scientific reasoning, judgment biases, and comprehension of regular science reporting.

### Is numeracy related to scientific reasoning?

Parallels between numeracy and scientific thinking have been discussed for more than 60 years. In the earliest known formal conceptualization of numeracy, the Crowther Report discussed numeracy as a “mirror image of literacy” and proposed understanding the scientific method as a possible component of numeracy (Ministry of Education, 1959, p. 270). More recent reports have also characterized numeracy skills as foundational prerequisites for being scientifically literate (National Academies of Sciences, 2016). Furthermore, some studies have noted that one must be able to use and apply mathematical knowledge to be able to engage in scientific reasoning and related problem-solving activities (e.g., proportional reasoning; Hilton & Hilton, 2016). However, specific theories about the nature of scientific reasoning have also been a topic of independent psychological inquiry for many years, including as part of Inhelder and Piaget’s (1958) theory of cognitive development. While that early theory assumed scientific reasoning was a single cognitive ability akin to a general latent trait with broad applications, later conceptualizations favored more multidimensional constructs (Opitz et al., 2017). For example, influenced by Newell and Simon’s (1972) theory of problem solving, Klahr and Dunbar (1988) developed an integrated model of scientific reasoning processes (*Scientific Discovery as Dual Search—SDDS*), which emphasized both domain-specific knowledge and general cognitive mechanisms.

More recently, scientific reasoning has been viewed as a concept that may contain multiple independent characteristics or skills (e.g., the eight skill model from Fischer et al., 2014; but see Opitz et al., 2017 for issues related to debates about the role of domain general vs. domain specific skills). Several modern theories emphasize independent roles of distinct general-purpose cognitive operations including induction, deduction, analogy, problem solving, and causal reasoning (Barz & Achimaş-Cadariu, 2016; Dunbar & Fugelsang, 2005; Kambeyo & Csapó, 2018). This view is generally consistent with broader modern conceptualizations of scientific reasoning defined primarily as the thinking and reasoning skills involved in (1) scientific inquiry, (2) experimentation, (3) evidence evaluation, (4) inference, and (5) argumentation (Zimmerman, 2000; but for related notions of scientific thinking and scientific discovery see also Bao et al., 2022; Díaz et al., 2023).

More than a dozen scientific reasoning tests have been developed since the turn of the century using a variety of test formats (e.g., closed vs. open ended; Opitz et al., 2017). Most of the modern tests are primarily intended to assess individual differences in skill achievement levels among high school and university students. However, the Scientific Reasoning Scale (SRS; Drummond & Fischhoff, 2017) is different because it was specifically designed and validated for broader use with educated adult samples (e.g., adults in industrialized countries). Recent studies have also directly examined the association between numeracy and scientific reasoning.^1^ For example, in a series of four studies, Drummond and Fischhoff (2017) found that numeracy was significantly correlated with scores on the SRS (*r* = 0.28, 0.36, and 0.40; Drummond & Fischhoff, 2017). Additionally, Drummond and Fischhoff (2019) conducted a series of studies to assess whether priming scientific reasoning skills might influence a person’s myside bias^2^ when evaluating scientific evidence. In one study, researchers utilized both the SRS and a numeracy test as priming conditions before participants evaluated scientific evidence. Although neither prime had a significant effect on participants’ myside bias, numeracy was found to be significantly related with SRS scores (*r* = 0.47). In another study that examined factors that might inhibit scientific reasoning skills, researchers developed two versions of the SRS; one that included concrete explanations (i.e., context rich) and one that was a context-free version of the same items. Results demonstrated that participants performed significantly worse on the decontextualized version, and numeracy was again found to be significantly correlated with SRS scores (*r* = 0.42; Bašnáková et al., 2021). Taken together, numeracy appears to be robustly and systematically related to scientific reasoning and related concepts among diverse adults. However, less is known about the cognitive mechanisms that link numeracy and scientific reasoning with regular science reporting and potential downstream judgment biases.

### Why do people misunderstand regular science reporting?

Recent research suggests that one common source of misunderstanding in regular science reporting may be *causal theory errors* (i.e., inferring or accepting a causal claim based on correlational evidence; Seifert et al., 2022). For example, results from Seifert et al. (2022) indicated that 63 percent of their participants erroneously accepted a causal claim based on correlational evidence alone. This finding accords with others that have shown similarly high rates of endorsing causal claims based on correlational evidence (Bleske-Rechek et al., 2015; Xiong et al., 2019; see also Adams et al., 2017; Norris et al., 2003). Given that science stories in the media have been linked to changes in both health-related behavior and knowledge (e.g., cancer screening decisions; Cram et al., 2003; Matthews et al., 2016; Stryker et al., 2008), these results seem to suggest that causal theory errors might lead to misunderstandings that could result in costly downstream attitude and judgment biases.

Recent research also suggests that interventions can reduce causal theory errors. For example, some studies have focused on the actions of science writers (e.g., journalists, researchers), suggesting that improving data visualization designs (Xiong et al., 2019) or including caveats (Bott et al., 2019) may help mitigate unwarranted perceptions of causality (see also Bratton et al., 2019, 2020; Sumner et al., 2014, 2016; Woloshin et al., 2009). Other studies have focused on interventions that directly aim to improve people’s reasoning when evaluating science studies (e.g., encouraging consideration of alternative causal theories; Seifert et al., 2022), which significantly reduced the occurrence of causal theory errors. The benefits of the reasoning intervention suggest that differences in cognitive strategies or knowledge can create differences in understanding and judgment. Additionally, there is some distant yet conceptually related evidence suggesting that differences in causal knowledge can simplify and inform related decision-making processes (Garcia-Retamero & Hoffrage, 2006; Garcia-Retamero et al., 2007). Furthermore, theories of causation have historically been linked to the philosophy of science (Cartwright, 2004; Woodward, 2005), which suggests that scientific reasoning and causal reasoning may be fundamentally related. These and other findings are consistent with the notion that both numeracy and scientific reasoning may be important skills that could reduce causal theory errors (Kuhn, 2012; Kuhn & Dean, 2004).

### Current studies

Despite potential connections, it remains unknown whether statistical numeracy and scientific reasoning skills are reliably related to causal understanding of regular science reporting. In accord with Skilled Decision Theory (Cokely et al., 2018), we hypothesized that more numerate people may be more likely to avoid causal theory errors primarily because they have acquired specialized scientific reasoning skills that help them accurately understand the secondary science reports. Therefore, a primary aim of the current studies was to provide the first direct test of a cognitive model, mapping the interrelations among numeracy, scientific reasoning, judgment biases, and causal theory errors in response to regular science reporting.

Study 1 presented participants with four real regular science report excerpts (e.g., an article from *CNN Health*), and tested a structural equation model of the relations between the variables of interest. Specifically, we hypothesized that scientific reasoning would partially mediate the relationship between numeracy and causal misunderstanding, and that these variables would predict risk perceptions and judgment biases. Study 2 then replicated the model from Study 1 and provided an out-of-sample cross-validation test. In addition, given that recent research suggests that more skilled decision makers may be better able to monitor their own understanding (i.e., confidence calibration; Ghazal et al., 2014), we extended the investigation in Study 2 to test the impact of participants’ metacognitive self-assessments on downstream judgment biases (i.e., susceptibility to overconfidence in science report comprehension). Consistent with Skilled Decision Theory, we hypothesized that overconfidence in one’s causal understanding would at least partially mediate the relations between numeracy and downstream judgment biases.

## Study 1

### Participants and procedure

The data were collected via Qualtrics in Spring 2023 using an undergraduate student sample from the University of Oklahoma. Participants signed up for the study as part of required course credit and were recruited through the university’s online recruiting system. The study was designed to take roughly one hour to complete, and the average completion time was about 40 min. Out of 276 total participants, 200 participants were retained for the analyses after excluding participants who did not meet IRB requirements, who completed less than 75% of the study, or who took less than half of the expected time to complete the study (i.e., less than 20 min; see Supplemental Materials for more details about the study exclusion criteria). Of the 200 participants, 160 (80%) identified as female, 32 (16%) identified as male, 2 (1%) identified as non-binary, and 6 (3%) selected “prefer not to say” or did not respond. Participants’ reported ages ranged between 18 and 33 years old (*M* = 19.4, *SD* = 1.8). For more detailed demographic information, see Table S1 in the Supplemental Materials.

Participants read four news article excerpts selected from online new sources (e.g., *CNN Health*), which were presented in a randomized order (Cosdon, 2022; Elliott, 2022; Kissell, 2022; Woodyatt, 2021).^3^ Each excerpt reported on scientific findings that had recently been published in peer-reviewed journals. The Flesch–Kincaid Readability and Grade Levels for all four article excerpts generally ranged from the high school graduate to college level (Kincaid et al., 1975; see Supplemental Materials for more information). The articles were selected because they each presented a correlational relationship between two variables that were apolitical in nature and discussed implications for health or other consequences (e.g., increased risk of dementia from watching too much TV). As such, each article presented an opportunity for a causal theory error. After each excerpt, participants answered questions related to causal misunderstanding (i.e., the extent to which they agreed correlational information presented in the article was causal), reported risk perceptions (i.e., how concerned they thought someone should be about the risk), and made judgments about multiple behavioral intentions (see below).

### Measures

#### Statistical numeracy

The Berlin Numeracy Test (BNT; Cokely et al., 2012) was used to assess numeracy and risk literacy. An example item is, “Imagine we are throwing a five-sided die 50 times. On average, out of these 50 throws how many times would this five-sided die show an odd number (1, 3 or 5)?” Following best-practice recommendations, this study included the BNT-S form, which includes four items from the BNT and three items from Schwartz et al. (1997), which provides increased sensitivity by allowing for a wider range of skill assessment. The sum of correct answers (0 – 7) was used as the statistical numeracy score.

#### Scientific reasoning

Scientific reasoning was assessed by the 11-item Scientific Reasoning Scale (Drummond & Fischhoff, 2017), which is an individual difference measure of the skills needed to evaluate the quality of scientific findings. Each item is comprised of a short scientific scenario followed by a statement that respondents must evaluate as either true or false. The sum of correct answers (0 – 11) was used as the scientific reasoning score.

#### Causal misunderstanding

Following Seifert et al. (2022), causal theory errors were operationalized as causal misunderstanding. Specifically, we measured participant’s responses to one item asking the extent to which participants agreed that the information presented in the article was causal (e.g., *According to the article, watching a lot of TV causes dementia*). Given that it is possible that people may have had prior relevant knowledge, we explicitly directed participants to respond solely based on the information provided in the article excerpts. The scale ranged from 1 (Not at all) to 6 (Strongly Agree). The mean rating of this statement across four article excerpts was used as the score for causal misunderstanding, with higher scores indicating more causal misunderstanding.^4^

#### Risk perceptions

Risk perceptions were measured with one item asking about the extent to which people should be concerned about the risk (e.g., *How concerned should someone who watches a lot of TV be about getting dementia?*). The scale ranged from 0 (Not at all concerned) to 6 (Extremely concerned). The mean rating of this item across four article excerpts was used as the score for risk perceptions.

#### Judgment biases

Judgment biases were measured using four items that assessed various possible judgments about behavioral intentions after reading each article excerpt (e.g., conditional intention to change one’s own behavior). The scale for each item ranged from 1 (Not at all) to 6 (Strongly Agree), with higher scores indicating more biased judgment. The four items are described in detail below:

***Personal.*** This item assessed the extent to which participants agreed that they would change their own behavior based on information presented in the article. An example item is, “*Given the information from the article, I would reduce the amount of TV that I watch to prevent dementia.*” The mean rating of this statement across four article excerpts was used as the score for “personal.”

***Recommendations.*** This item assessed the extent to which participants agreed that they would recommend a change in behavior to their friends and family. An example item is, “*Given the information from the article, I would recommend that my friends and family reduce the amount of TV that they watch.*” The mean rating of this statement across four article excerpts was used as the score for “recommendations.”

***Social Media.*** This item assessed the extent to which participants agreed that they would share information about the risk on social media. An example item is, “*Given the information from the article, I would share on social media that it is important to reduce the amount of TV watched in order to prevent dementia.*” The mean rating of this statement across four article excerpts was used as the score for “social media.”

***Policy.*** This item assessed the extent to which participants agreed that they would redirect funding within a local community. An example item is, “*A local community has a risk communication program that aims to improve people’s health. They are considering redirecting some funding for HIV/AIDS risk communication toward risk communication that gives a warning about the relationship between watching TV and dementia. Based on the information from the article, they should choose to redirect the funding.*” The mean rating of this statement across four article excerpts was used as the score for “policy.”

## Results and analyses

**Fig. 1:**
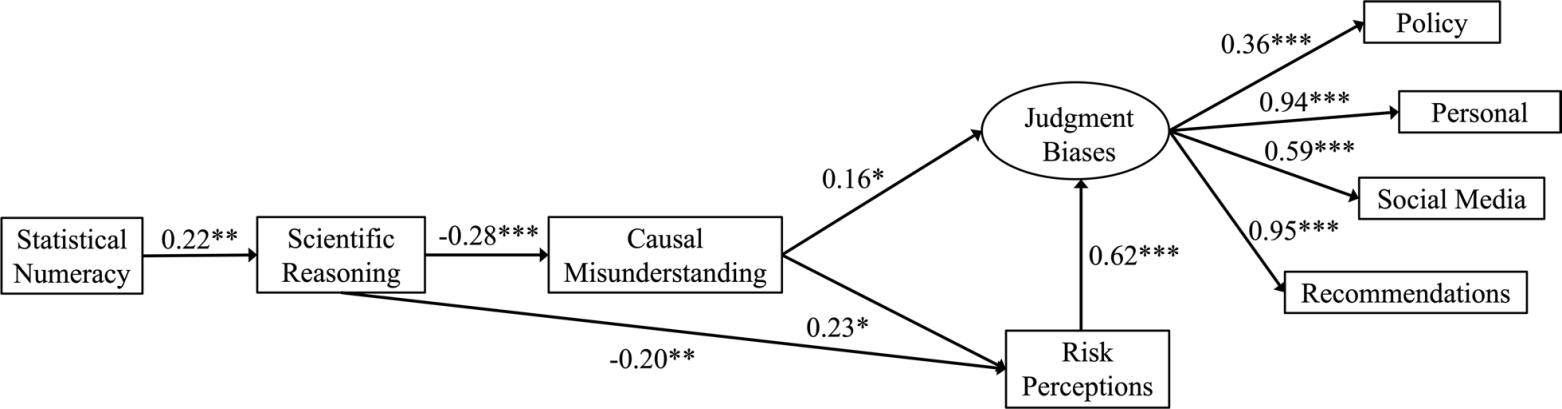
Structural equation model with causal misunderstanding. *Note*. Standardized coefficients are reported. *p < .05, **p < .01, ***p < .001. Model estimated using the *lavaan* package in R (Rosseel, 2012)

All statistical analyses were completed in R version 4.3.0. Base R packages were used to compute descriptive statistics and correlations for all Study 1 variables (Table 1). To test relationships between numeracy, scientific reasoning, causal misunderstanding, and downstream consequences, a structural model was estimated using the *lavaan* package (Rosseel, 2012), and a latent factor of judgment biases was estimated and included in the structural model (Fig. 1). In the first model tested, all pathways between independent variables and outcomes were freely estimated. Figure 1 displays the final model with only significant paths included. Furthermore, relevant indirect effects within the model were tested and presented with 95% confidence intervals (CI) estimated using 1000 bootstrap samples (Table 2).

**Table 1:** Descriptive statistics and correlations for study 1 variables

Variable	*M*	*SD*	1	2	3	4	5	6	7	8
1. BNT-S	3.14	1.67								
2. SRS	5.68	2.45	.22**							
3. Causal misunderstanding	3.59	1.06	− .08	− .28**						
4. Risk perceptions	3.40	0.85	.01	− .27**	.29**					
5. Judgment biases	3.42	0.74	.02	− .25**	.38**	.66**				
6. Social media	2.80	1.11	.01	− .25**	.29**	.51**	.76**			
7. Recommendations	3.87	0.85	.05	− .19**	.32**	.63**	.87**	.55**		
8. Personal	3.83	0.85	.02	− .23**	.30**	.61**	.86**	.56**	.89**	
9. Policy	3.19	1.05	− .02	− .10	.27**	.32**	.62**	.20**	.35**	.30**

*p < .05, **p < .01

**Table 2:** Total indirect effects (standardized coefficients) for key variables in Study 1

Paths	Estimate	SE	Bootstrapped 95% CI
(1) Numeracy → scientific reasoning → *causal misunderstanding*	− .06*	.03	[− .12, − .02]
(1) Numeracy → scientific reasoning → *risk perceptions* (2) Numeracy → scientific reasoning → causal misunderstanding → *risk perceptions*	− .06*	.02	[− .11, − .02]
(1) Numeracy → scientific reasoning → risk perceptions → *judgment biases* (2) Numeracy → scientific reasoning → causal misunderstanding → *judgment biases* (3) Numeracy → scientific reasoning → causal misunderstanding → risk perceptions → *judgment biases*	− .05**	.02	[− .09, − .02]

Estimate reflects the total indirect effect from multiple paths for each dependent variable. Effects were estimated with 1000 bootstrap samples

SE, standard error; CI, confidence interval

*p < .05, **p < .01

As shown in Fig. 1, structural equation modeling (SEM) suggested that more numerate people were less likely to misunderstand correlational information as causal specifically because they tended to be better at scientific reasoning. In accord with accepted standards for model evaluation (Hu & Bentler, 1999), this model demonstrated good fit, *χ*^2^_18_ = 29.17, *p* = 0.046, CFI = 0.98, TLI = 0.97, RMSEA = 0.06 [0.01, 0.09], SRMR = 0.05. Specifically, statistical numeracy significantly predicted scientific reasoning (*β* = 0.22), which in turn significantly predicted causal misunderstanding (*β* = −0.28). Causal misunderstanding also partially mediated the relationship between scientific reasoning and risk perceptions. Furthermore, causal misunderstanding mediated the relationship between scientific reasoning and judgment biases (see Fig. 1).

As shown in Table 2, an analysis of indirect effects indicated that numeracy exerted a significant indirect effect on causal misunderstanding (− 0.06, 95% CI [− 0.12, − 0.02]) as well as on risk perceptions (− 0.06, 95% CI [− 0.11, − 0.02]). Finally, numeracy exerted a significant indirect effect on judgment biases, through scientific reasoning, causal misunderstanding, and risk perceptions (− 0.05, 95% CI [− 0.09, − 0.02]).

## Study 1 Discussion

Results from Study 1 indicate that more numerate people were indeed less likely to misunderstand regular science reporting as compared to less numerate people. SEM results suggested that more numerate people were less likely to misconstrue correlational information as causal, which in turn led to fewer downstream biases (i.e., less biased risk perceptions and judgments). These observed benefits of numeracy followed because more numerate people had previously acquired specialized scientific reasoning skills. Theoretically, this evidence suggests that one reason more numerate people may become more knowledgeable and informed decision-makers is that they may be more likely to acquire some additional risk literacy skills (i.e., scientific reasoning) that may often help facilitate risk comprehension and knowledge acquisition.

While these findings were largely consistent with anticipated results, we had specific concerns about two primary limitations of the current study. First, results suggested that some participants may not have complied with instructions (e.g., 67% of participants failed one of two attention checks). This potentially careless responding could have implications for the reliability and interpretability of the observed model fit (see Voss, 2024 for a brief review), which suggested that replication and cross-validation was merited. Second, it is possible that using a strength of agreement Likert scale to assess causal misunderstanding may have weakened the psychometric precision of the variable.^5^ Although using a Likert scale has some advantages (e.g., wider range of response options), measuring causal misunderstanding in this way made it more difficult to precisely or unequivocally estimate causal theory errors. Furthermore, given the influential role of knowledge outlined in other recent research (e.g., Cho et al., 2023), it also seemed likely that, provided sufficient psychometric sensitivity, causal misunderstanding could fully mediate the relationship between scientific reasoning and downstream consequences (i.e., risk perceptions and judgment biases), which we also felt merited additional testing.

## Study 2

To begin to address potential measurement limitations of Study 1, Study 2 included an updated measure of causal misunderstanding and an additional test of a metacognition bias (i.e., overconfidence), which was investigated using two of the four article excerpts from Study 1. Specifically, in Study 2, causal misunderstanding was measured via a single binary choice item, allowing for a more discrete measure of causal theory errors. In addition, Study 2 aimed to address previous research suggesting that metacognitive biases may affect people’s ability to acquire new information because they may limit people’s ability to evaluate their own comprehension (e.g., overconfidence; Scopelliti et al., 2015; Ybarra, 2018). Recent research suggests that more skilled decision-makers (i.e., more numerate) may often use their skills to develop a more integrated, representative understanding of the relevant information, which typically allows them to better monitor their own understanding (e.g., more calibrated self-evaluations and less overconfidence bias; Cokely et al., 2018; Ghazal et al., 2014; Ybarra, 2018). To test these potential relations, Study 2 included a measure of confidence in one’s causal comprehension, which was used in concert with the causal misunderstanding item to include overconfidence in the cognitive model. Following previous research, we hypothesized that overconfidence would at least partially mediate the relations between numeracy, scientific reasoning, risk perceptions, and judgment biases.

### Participants and procedure

The data were collected via Qualtrics in Fall 2023 using an undergraduate student sample from the University of Oklahoma. Participants signed up for the study as part of required course credit and were recruited through the university’s online recruiting system. The study was designed to take roughly 30 min to complete, and the average completion time was about 20 min. Out of 401 total cases, 342 were used for the analyses after excluding participants who did not meet IRB requirements, who completed less than 75% of the study, or who took less than half of the expected time to complete the study (i.e., less than 10 min; see Supplemental Materials for more details about the study exclusion criteria). Of the 342 participants, 269 (78.6%) identified as female, 71 (20.8%) identified as male, and 2 (0.6%) identified as non-binary. Participants’ reported ages ranged between 18 and 34 years old. See Table S1 in the Supplemental Materials for more detailed demographic information.

Participants read two news article excerpts in a randomized order (both excerpts were also used in Study 1; Cosdon, 2022; Kissell, 2022). Each excerpt reported scientific findings published in peer-reviewed journals and presented an opportunity to make a causal theory error. After each excerpt, participants answered a question related to causal misunderstanding (i.e., whether they thought the correlational information presented in the article was causal), confidence, risk perceptions (i.e., how concerned they thought someone should be about the risk), and multiple judgments about behavioral intentions following the protocol in Study 1.

### Measures

All measures from Study 1 were included in Study 2. This includes: (1) *Statistical Numeracy*, (2) *Scientific Reasoning*, (3) *Causal Misunderstanding*, (4) *Risk Perceptions*, and (5) *Judgment Biases*. However, in this study, causal misunderstanding was measured by a single binary item (True/False) and one additional variable was measured: *Confidence*.

#### Causal misunderstanding

Causal misunderstanding was measured by one item which asked participants whether they thought the information presented in the article was causal (e.g., *According to the article, watching a lot of TV causes dementia*). Respondents were required to evaluate the statement as true or false. Correct answers (false) were scored as “1”, while incorrect answers (true) were scored as “0”.

#### Confidence

Confidence was measured with an item asking the degree to which participants believe their causal judgment was accurate (i.e., *How confident are you that your above judgment is accurate?*). The scale ranged from 0% confident to 100% confident in increments of 10%.

#### Causal misunderstanding: overconfidence

The score for this variable was calculated as a participant’s causal misunderstanding score subtracted from their confidence score. Confidence scores were converted from whole numbers to decimals for the calculation (e.g., 100% confident = 1.0, 90% confident = 0.9). Scores for this variable ranged from −1 (highly underconfident) to 1 (highly overconfident), where a score of 0 indicated neither over nor underconfident. For example, someone who scores a 0 on the causal misunderstanding item (incorrect) but is 90% confident that their answer was correct would receive a score of 0.9 (i.e., 0.9–0 = 0.9), indicating a high degree of overconfidence in their judgment accuracy.

## Results and analyses

**Fig. 2:**
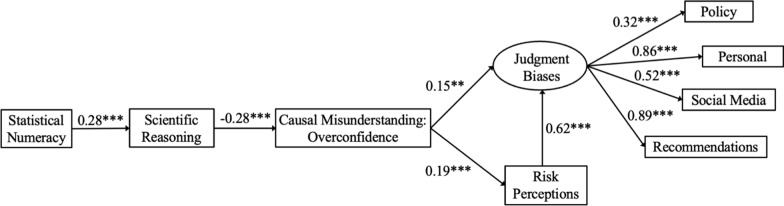
Structural equation model with overconfidence calculated as a function of causal misunderstanding. *Note.* Standardized coefficients are reported. *p < .05, **p < .01, ***p < .001. Model estimated using the *lavaan* package in R (Rosseel, 2012)

All statistical analyses were completed in R version 4.3.0. Base R packages were used to compute descriptive statistics and correlations for all Study 2 variables (Table 3). To test relationships between numeracy, scientific reasoning, causal misunderstanding overconfidence,^6^ and downstream judgment biases, a structural modeling approach was again used, and a latent trait of judgment biases was estimated in the structural model (Fig. 2). In the first model tested, all pathways between independent variables and outcomes were freely estimated. Figure 2 displays the final model with only significant paths included. Relevant indirect effects within the model were tested and presented with 95% CI estimated using 1000 bootstrap samples (Table 4).

**Table 3:** Descriptive statistics and correlations for Study 2 variables

Variable	*M*	*SD*	1	2	3	4	5	6	7	8
1. BNT-S	3.72	1.74								
2. SRS	5.85	2.51	.28**							
3. Causal misunderstanding: overconfidence	0.29	0.43	− .12*	− .28**						
4. Risk perceptions	3.88	1.03	.03	− .11*	.19**					
5. Judgment biases	3.64	0.79	.05	− .23**	.26**	.55**				
6. Social media	2.83	1.19	− .02	− .27**	.21**	.31**	.73**			
7. Recommendations	4.21	1.02	.07	− .14*	.23**	.60**	.83**	.43**		
8. Personal	4.10	1.05	.12*	− .12*	.23**	.53**	.85**	.49**	.77**	
9. Policy	3.39	0.97	− .04	− .13*	.09	.22**	.56**	.17**	.27**	.28**

*p < .05, **p < .01

**Table 4:** Total indirect effects (standardized coefficients) for key variables in Study 2

Paths	Estimate	SE	Bootstrapped 95% CI
(1) Numeracy → scientific reasoning → *causal misunderstanding: overconfidence*	− .08***	.02	[− .12, − .04]
(1) Numeracy → scientific reasoning → causal misunderstanding: overconfidence → *risk perceptions*	− .02*	.01	[− .03, − .01]
(1) Numeracy → scientific reasoning → causal misunderstanding: overconfidence → *judgment biases* (2) Numeracy → scientific reasoning → causal misunderstanding: overconfidence → risk perceptions → *judgment biases*	− .02**	.01	[− .04, − .01]

Estimate reflects the total indirect effect from multiple paths for each dependent variable. Effects were estimated with 1000 bootstrap samples

SE, standard error; CI, confidence interval

*p < .05, **p < .01, ***p < .001

As shown in Fig. 2, structural equation modeling indicated numeracy was related to reduced overconfidence in causal misunderstanding because more numerate people tended to be better at scientific reasoning (i.e., less overconfidence as a function of accuracy of understanding). The model exhibited acceptable fit, *χ*^2^_19_ = 45.40, *p* = 0.001, CFI = 0.96, TLI = 0.94, RMSEA = 0.06 [0.04, 0.09], SRMR = 0.06, such that statistical numeracy predicted scientific reasoning (*β* = 0.28), which in turn predicted overconfidence in causal misunderstanding (*β* = −0.28). Additionally, structural equation modeling revealed that the improved causal misunderstanding: overconfidence variable fully mediated the relationship between scientific reasoning and all downstream judgment biases (see Fig. 2). As shown in Table 4, an analysis of indirect effects confirmed that numeracy exerted a significant indirect effect on causal misunderstanding: overconfidence (− 0.08, 95% CI [− 0.12, − 0.04]) and risk perceptions (− 0.02, 95% CI [− 0.03, − 0.01]). Further, numeracy exerted a significant indirect effect on judgment biases, through scientific reasoning, causal misunderstanding: overconfidence, and risk perceptions (− 0.02, 95% CI [− 0.04, − 0.01]).

## Study 2 Discussion

Study 2 verified and extended findings from Study 1 using refined assessments, including a metacognitive judgment on the causal theory error question. Consistent with primary findings from Study 1, results from Study 2 again suggested that more numerate people were less likely to misinterpret correlational information as causal, and in turn, were less likely to exhibit downstream judgment biases. Once again, the structural equation model indicated that the benefits of numeracy primarily resulted because more numerate people had previously acquired specialized scientific reasoning skills, which fully explained the relations between numeracy and downstream judgment biases. By introducing a new variable (i.e., causal misunderstanding: overconfidence), Study 2 also provided novel evidence on the role of metacognition—i.e., overconfidence in one’s own understanding of regular science reporting. As anticipated, overconfidence in science report comprehension fully mediated the relationship between scientific reasoning and all downstream judgment biases (i.e., people who were more overconfident in their understanding were more likely to express judgment biases).

## General discussion

The current findings suggest that more numerate people may typically be more likely to accurately evaluate and understand at least some important elements of regular science reporting (i.e., less likely to interpret correlational evidence as causal). These studies also appear to be the first to test a cognitive model linking numeracy, scientific reasoning, causal theory errors, and downstream judgment biases with people’s interpretations of regular science reporting. Findings indicate that more numerate people may be less likely to make causal theory errors when reviewing regular science reporting primarily because numerate people are more likely to have acquired and applied specialized scientific reasoning skills. Study 2 replicated and extended these findings, further revealing that numerate people’s scientific reasoning skills also helped them gain more metacognitive insight into their own understanding (i.e., reduced overconfidence). In turn, numerate people’s ability to accurately evaluate and understand the regular science reporting promoted more informed and less biased judgments (e.g., risk perceptions, behavioral intentions, and policy preferences). Overall, results reveal some potential obstacles that may make independent (unaided) evaluation of regular science reporting difficult for many people. Results also shed new light on one reason numerate individuals may independently acquire some of the knowledge they typically rely on to make more informed and less biased judgments and decisions.

### Risk literacy is not (just) statistical numeracy

The theoretical construct of *Risk Literacy* has been defined as “the ability to evaluate and understand risk” (Cokely et al., 2018; see also Cokely et al., 2012, 2013, 2014; Feltz et al., 2022; Feltz & Cokely, 2024a; Garcia-Retamero & Cokely, 2017; Gigerenzer, 2012, 2015; Reyna & Brainerd, 2023). This conceptualization suggests that risk literacy skills are not identical to statistical numeracy skills, even though the two sets of skills tend to be robustly linked (e.g., statistical numeracy skills fundamentally involve practical skills for probabilistic and inductive reasoning; Cokely et al., 2012, 2013, 2014, 2018). A related conceptual view has recently been advanced by Aven (2024) suggesting that risk literacy should not be too narrowly construed as probabilistic reasoning.^7^ Instead, Aven argues for a broader concept of risk literacy that includes other skills present in practitioners, scientists, and experts in fields of risk analysis, risk science, and decision analysis. For example, Aven noted that a broader view of risk literacy should include skills related to other fundamental concepts of science (e.g., distinguishing correlation from causation; Aven, 2020), which are often elements of expert risk analysis. Accordingly, the current studies provide some of the first evidence linking both numeracy and science reasoning with risk literacy and skilled decision-making more generally.

The current findings are also consistent with evidence revealing that more numerate people tend to independently acquire other valuable reasoning skills, including more advanced graph literacy and metacognitive skills, which also promote risk literacy in ways that mediate the influence of numeracy (Garcia-Retamero & Cokely, 2013, 2017; Ghazal et al., 2014). However, research suggests numeracy skills may be prerequisites for the acquisition of science reasoning skills, which is not necessarily the case for metacognitive and graph literacy skills (National Academies of Sciences, 2016). If correct, this likely has notable implications for the order of training programs used to develop risk literacy skills (e.g., a broad foundation in statistical numeracy skills should come before scientific reasoning training).

## Limitations

One limitation of the current investigation is that it employed a relatively narrow band of materials and participants. As such, future investigations may want to consider testing a wider range of articles (e.g., other publishers and formats; more and less controversial or familiar topics), across multiple domains (e.g., other than health-related domains; Dhami et al., 2004). Researchers may also want to consider recruiting more representative and diverse samples (e.g., other than US public college students). That said, given the nature of the mechanisms involved in the current research, it seems likely to us that findings will generalize to a meaningful extent (i.e., key findings are primarily about the configural relations between skills, reasoning, and interpretations). Still, it is possible that other factors such as prior knowledge^8^ or biases could interact in unusual ways. For example, motivated reasoning biases might possibly overshadow the benefits of numeracy or science reasoning in special situations, such as those dealing with controversial risks (e.g., guns, climate change) that involve entrenched biases, extreme worldviews, and other conflicts of interest (Cho et al., 2023; Kahne & Bowyer, 2017; Roh et al., 2015; Taber & Lodge, 2006). Furthermore, it is difficult to know why participants may or may not have rejected the causal claims. For example, a participant may not have conflated correlation and causation, but might have still rejected the causal claim based on their personal beliefs or past experiences.

Another potential limitation of the current investigation is that the direct effect of numeracy on causal misunderstanding was not significant in Study 1. The lack of a significant direct relation in Study 1 appears to be explained by limits of the psychometric sensitivity of the misunderstanding variable that was used. The sensitivity issue was addressed in Study 2 by modifying the misunderstanding variable, which then demonstrated a small but significant relationship between numeracy and overconfidence in one’s causal understanding (see correlation matrix in Table 3). The indirect effect linking numeracy to causal misunderstanding via scientific reasoning skills was also significant in both Study 1 and 2. Taken together, the overall direct effect of numeracy appears to be reliable yet modest. Nevertheless, even if the relationship only confers small improvements for understanding on each specific science report, these benefits may be likely to add up over time. Given the evidence that overall engagement with science news has increased in recent years (Hendriks et al., 2020; National Science Board, 2018; Pavelle & Wilkinson, 2020; Saks & Tyson, 2022), if numerate people acquire just one extra fact from each of the articles they read, some numerate readers might be likely to learn thousands of additional decision-relevant facts each year.

### Risk literacy difficulty analysis

Given the modest yet consistent link between numeracy, scientific reasoning, and causal misunderstanding that was observed, we conducted a Risk Literacy Difficulty Analysis to further investigate potential practical implications of these relations (Allan, 2021).^9^ Using the methodology developed by Allan (2021), we estimated how difficult each task was in terms of (a) the proportion of US adults likely to misunderstand similar science reports, and (b) how much numeracy skill would typically be needed to likely avoid misinterpretations of similar science reports. Accordingly, using data from Study 2, we conducted a Risk Literacy Difficulty Analysis for each of the two article excerpts (i.e., TV and dementia; vitamin D and COVID-19 severity). The Risk Literacy Difficulty Analysis suggests that the minimum BNT-S score likely needed for accurate causal interpretation of the TV and dementia article (i.e., correctly rejecting the causal claim) was 4.4 out of 7. Using the US adult norms provided, this suggests that around 70% of the general US adult population may be likely to make causal theory errors when evaluating this science report. The vitamin D and COVID-19 severity article produced similar estimates, and suggests that around 75% of the general US adult population may be likely to make a causal theory error. Assuming the results generalize to other similar science reports, the Risk Literacy Difficulty Analysis suggests that more than half of all adult residents in the USA may be likely to misunderstand information in regular science reports like the ones used in the current study.

## Conclusions

The current work sheds new light on how numerate people may become more informed and less biased decision-makers. Consistent with Skilled Decision Theory, findings suggest more numerate people (a) tend to acquire specialized scientific reasoning skills that promote the ability to evaluate and understand risk information (i.e., risk literacy), and (b) use their risk literacy skills to both learn from, and avoid being misled by, regular science reporting. Unfortunately, the results from the current studies suggest that for many people the information provided in secondary science reports may be more confusing than it is informative. One way to address this disparity may be to improve science reporting standards (e.g., how researchers and journalists report findings). However, given potential conflicts of interest (Feltz & Cokely, 2024b), inconsistency in adherence to professional standards (Garcia-Retamero & Cokely, 2017; Garcia-Retamero et al., 2020), and the many growing threats from disinformation and misinformation (Van der Linden, 2023), future research should also develop specialized risk literacy training programs that help diverse people independently and accurately interpret science information on their own. Taken altogether, our findings suggest risk literate people may generally be less biased decision-makers because their skills help them understand and accrue knowledge from reputable science reporting.

## Supplementary Information

Additional file 1.

## Data Availability

The datasets and materials used in the current studies are available in the Open Science Framework repository, [https://osf.io/pyj9t/]. This manuscript does not contain any preregistered experiments.

## Abbreviations

BNT: Berlin numeracy test
BNT-S: Berlin numeracy test-Schwartz
CFI: Comparative fit index
CI: Confidence interval
CNN: Cable news network
RMSEA: Root mean square error of approximation
SDDS: Scientific discovery as dual search
SE: Standard error
SEM: Structural equation modeling
SRMR: Standard root mean square residual
SRS: Scientific reasoning scale
TLI: Tucker–Lewis index

## Footnotes

1 Other related concepts such as science literacy have also been shown to be related to numeracy, in the context of complex medical screening decisions (Petrova et al., 2016, 2019) and perceptions of immigration (Savadori et al., 2022).

2 Myside refers to an individual’s opinion or position on a particular issue or topic. Therefore, myside bias refers to an individual’s tendency to generate and evaluate evidence in ways that favor their own existing positions and beliefs (Drummond & Fischhoff, 2019; Toplak & Stanovich, 2003).

3 Study 1 included a between participants experimental manipulation (i.e., drawing attention to the correlational nature of the study by modifying the phrasing of information about “risks” by replacing it with information about “correlations”). This experimental manipulation was ultimately not significant. Therefore, all following analyses are collapsed across conditions. See Supplemental Materials for more information about the manipulation.

4 Two items were originally used to assess causal misunderstanding in Study 1. However, one item was dropped as the wording seemed to elicit confusion.

5 See the general discussion for a detailed discussion of another potential limitation, namely, the non-significant direct effect of numeracy, which also appears to have been the result of a psychometric measurement limitation (see also Study 2).

6 *Causal misunderstanding overconfidence*, as discussed in the results, refers to the combined measure of participants’ causal misunderstanding score and confidence score (e.g., higher scores demonstrate overconfidence as a function of accuracy of understanding; see Method section “Causal Misunderstanding: Overconfidence” for details).

7 Aven suggested the concept of risk literacy should not be conflated with probabilistic reasoning, rejecting the notion that risk literacy should be primarily defined by the Knightian dichotomy between risk and uncertainty (e.g., where risk is strictly viewed as known, objective probabilities; Knight, 1921).

8 Prior knowledge was not measured in the current studies and theoretically there may be other campaigns and research findings that may be relevant (e.g., Ihira et al., 2022; Inoue et al., 2023; Vallance et al., 2018). However, to limit the role of prior knowledge, the causal judgment and behavioral intentions questions explicitly asked participants to respond solely based on the information provided in the brief article excerpts. Nevertheless, it may still be the case that participants utilized prior knowledge, which may have shaped their responses to causal judgments and downstream behavioral intentions.

9 Using data from a probability-based representative sample of US adults, this methodology provides a means to compare the Risk Literacy Difficulty Level of different tasks. Specifically, the method provides an estimate of the probability of misunderstanding among the general US adult population, as well as a means to estimate the minimum score on the Berlin Numeracy Test typically required for someone to be “more likely than not” to avoid errors and biases, defined as achieving at least 50% task accuracy; see Allan, 2021 for more details). Although estimating accuracy is known to depend on multiple model assumptions and research design factors (e.g., how representative are tasks; how psychometrically reliable are dependent measures), initial investigations of this method indicate it is likely to have robust predictive validity, as evidenced in out-of-sample cross-validation tests.
