# Supplementary material for: Numerate people are less likely to be biased by regular science reporting: the critical roles of scientific reasoning and causal misunderstanding
Source: Cogn Res Princ Implic. 2025 Jun 15;10:32. doi: 10.1186/s41235-025-00641-6 (PMC12167740; doi:10.1186/s41235-025-00641-6)
Supplement: Supplementary file 1 — Additional file 1. [file 41235_2025_641_MOESM1_ESM.docx]

**Supplemental Materials**

**Explanation of Study Exclusion Criteria**

Study 1:

Started with 276 participants

- Removed anyone who reported age as less than 18 (n = 1)

- Removed anyone who completed less than 75% of the study (n = 20)

- 13 of the 20 participants completed 20 percent or less of the study

- 9 of the 13 completed 0 percent of the study

- Removed anyone who took less than 20 minutes (half of expected time) (n = 55)

- Final participant total: **200 participants**

Study 2:

Started with 401 participants

- Removed anyone who did not sign the informed consent (n = 3)

- Removed anyone who reported age as less than 18 (n = 2)

- Removed anyone who completed less than 75% of the study (n = 22)

- 18 of 22 participants completed 25 percent or less of the study

- 16 of the 18 completed only 6 percent

- Removed anyone who took less than 10 minutes (half of expected time) (n = 32)

- New participant total: **342 participants**

**Study 1 Manipulation**

Study 1 included a between participants experimental manipulation, where we aimed to draw attention to the correlational nature in the passages by making a slight modification to the phrasing of information. For this manipulation, we replaced each passage’s mention of “risks” with a mention of “correlations,” in order to directly acknowledge the correlational nature of the information. A series of t-tests demonstrated that our experimental manipulation was not significant for the causal misunderstanding item (*t*(195) = -1.26, *p* = 0.21), risk perceptions (*t*(192) = -0.94, *p* = 0.35), and the four judgment bias variables (policy, *t*(182) = -0.37, *p* = 0.71; personal, *t*(192) = 0.62, *p* = 0.54; recommendations, *t*(186) = 1.12, *p* = 0.27; social media, *t*(196) = 0.14, *p* = 0.89). Therefore, all following analyses for Study 1 were collapsed across conditions. The R code for all t-tests is available on the OSF repository in the provided R script.

**Flesch-Kincaid Grade Level and Readability Ease Scores (from goodcalculators.com)**

TV and Dementia Article (Kissell, 2022): Grade Level – 12.8, Readability Ease – 35.9

Vitamin D and COVID-19 Article (Cosdon, 2022): Grade Level – 13, Readability Ease – 30.1

Dairy and Heart Disease Article (Woodyatt, 2021): Grade Level – 16.6, Readability Ease – 31.6

Exercise and Premature Death Article (Elliot, 2022): Grade Level – 11.3, Readability Ease – 45.3

**Table S1**

*Study 1 and Study 2 Demographics*

|  | **Study 1 Respondents n (%)** | **Study 2 Respondents n (%)** |
| --- | --- | --- |
| **Gender** |  |  |
| Female | 160 (80.0%) | 269 (78.7%) |
| Male | 32 (16.0%) | 71 (20.8%) |
| Non-binary | 2 (1.0%) | 2 (0.6%) |
| Prefer not to say/NA | 6 (3.0%) | 0 (0.0%) |
| **Age** |  |  |
| 18 to 24 | 192 (96.0%) | 337 (98.5%) |
| 25 to 34 | 3 (1.5%) | 5 (34.2%) |
| NA | 5 (2.5%) | 0 (0.0%) |
| **Race** |  |  |
| White | 140 (70.0%) | 241 (70.5%) |
| Black or African American | 18 (9.0%) | 21 (6.1%) |
| American Indian or Alaska Native | 13 (6.5%) | 14 (4.1%) |
| Asian | 14 (7.0%) | 45 (13.2%) |
| Native Hawaiian or Pacific Islander | 0 (0.0%) | 0 (0.0%) |
| Other | 12 (6.0%) | 20 (5.8%) |
| NA | 3 (1.5%) | 1 (0.3%) |
| **Hispanic** |  |  |
| No | 162 (81.0%) | 286 (83.6%) |
| Yes | 35 (17.5%) | 55 (16.1%) |
| NA | 3 (1.5%) | 1 (0.3%) |
| **Political Party** |  |  |
| Republican | 67 (33.5%) | 110 (32.2%) |
| Democrat | 48 (24.0%) | 74 (21.6%) |
| Independent | 32 (16.0%) | 52 (15.2%) |
| Another Party | 3 (1.5%) | 1 (0.3%) |
| No Preference | 35 (17.5%) | 80 (23.4%) |
| Prefer not to respond/NA | 15 (7.5%) | 25 (7.3%) |
| **TOTAL** | **200** | **342** |

**Article Excerpts**

**Article 1 (Included in Studies 1 & 2)**

Kissell, C. (2022, August 26). *Watching too much TV linked to increased risk of dementia*. Money Talks News. [https://www.moneytalksnews.com/watching-too-much-tv-linked-to-increased-risk-of-dementia/](https://nam12.safelinks.protection.outlook.com/?url=https%3A%2F%2Fwww.moneytalksnews.com%2Fwatching-too-much-tv-linked-to-increased-risk-of-dementia%2F&data=05%7C02%7Cjinana%40clemson.edu%7Ce405a3419d964bf96d3f08dc4a0f4b8f%7C0c9bf8f6ccad4b87818d49026938aa97%7C0%7C0%7C638466676743641052%7CUnknown%7CTWFpbGZsb3d8eyJWIjoiMC4wLjAwMDAiLCJQIjoiV2luMzIiLCJBTiI6Ik1haWwiLCJXVCI6Mn0%3D%7C0%7C%7C%7C&sdata=KFtjWNmB9Cn57H92OX5t3wCiWKiYNTtyi1MCaxXE%2BTI%3D&reserved=0)

**Watching Too Much TV Linked to Increased Risk of Dementia**

With age comes the temptation to engage in watching hours of TV and other passive activities. But a new study suggests that such passivity might put our brains at risk.

Being 60 years of age or older and engaging in long periods of sitting passively may increase the risk of developing dementia, according to researchers at the University of Southern California and the University of Arizona.

However, the risk diminishes for those who sit while actively engaging the mind, such as when using the computer. The findings were published in the journal Proceedings of the National Academy of Science.

In a press release summarizing the study findings, study author David Raichlen, professor of biological sciences and anthropology at USC, says: “We know from past studies that watching TV involves low levels of muscle activity and energy use compared with using a computer or reading. And while research has shown that uninterrupted sitting for long periods is linked with reduced blood flow in the brain, the relatively greater intellectual stimulation that occurs during computer use may counteract the negative effects of sitting.”

The researchers analyzed self-reported data from the U.K. Biobank, a large biomedical database of participants throughout the United Kingdom. Specifically, they looked at data on more than 145,000 participants who were age 60 or older and were followed for about 12 years, on average.

The researchers found that spending time watching TV was associated with an increased risk of dementia even among people who are physically active. By contrast, spending time using a computer was associated with a reduced risk of developing dementia.

**Article 2 (Included in Studies 1 & 2)**

Cosdon, N. (2022, February 3). *Vitamin D Deficiency Increases Risk of Severe or Fatal COVID-19.*ContagionLive. [https://www.contagionlive.com/view/vitamin-d-deficiency-increases-risk-of-severe-or-fatal-covid-19](https://nam12.safelinks.protection.outlook.com/?url=https%3A%2F%2Fwww.contagionlive.com%2Fview%2Fvitamin-d-deficiency-increases-risk-of-severe-or-fatal-covid-19&data=05%7C02%7Cjinana%40clemson.edu%7Ce405a3419d964bf96d3f08dc4a0f4b8f%7C0c9bf8f6ccad4b87818d49026938aa97%7C0%7C0%7C638466676743626409%7CUnknown%7CTWFpbGZsb3d8eyJWIjoiMC4wLjAwMDAiLCJQIjoiV2luMzIiLCJBTiI6Ik1haWwiLCJXVCI6Mn0%3D%7C0%7C%7C%7C&sdata=DNM%2FCVV0vgDN230AGxMR9Wdh1JrWs4st2VoUNUXYqKc%3D&reserved=0)

**Vitamin D Deficiency Increases Risk of Severe or Fatal COVID-19**

Vitamin D is crucial to maintain bone health and facilitate immune system function. Early in the pandemic, health officials encouraged the public to take vitamin D supplements to boost immune response and potentially protect against COVID-19.

Now, studies are increasingly demonstrating that low vitamin D levels pose an increased risk of COVID-19 infection and death. One study, published today in PLOS ONE, is among the first to analyze vitamin D levels prior to COVID-19 infection.

Jointly led by Bar-Ilan University and Galilee Medical Center, the study looked for any correlation between pre-infection serum 25-hydroxyvitamin D (25(OH)D) level and COVID-19 severity and mortality. Investigators analyzed hospital records of individuals admitted to the Galilee Medical Center in Nahariya, Israel from April 7, 2020-February 4, 2021. Included subjects had a positive PCR COVID-19 test and 25(OH)D levels documented 14-730 days prior to their infection.

The investigators grouped patients by disease severity and level of 25(OH)D. Vitamin D levels were categorized as deficient, insufficient, adequate, or high-normal. They determined COVID-19 disease severity using multivariable regression analysis. Utilizing a cosinor model, the investigators isolated the influence of a sinusoidal pattern of seasonal 25(OH)D fluctuations.

Of the 1176 patients included in the study, 253 had a record of their 25(OH)D level before COVID-19 infection. Patients with a vitamin D deficiency, defined as < 20 ng/mL, were 14 times more likely to have severe or fatal COVID-19 disease than patients with 25(OH)D ≥ 40 ng/mL.

Overall, a low vitamin D status was present in 87.4% of patients with severe or critical COVID-19, but only 34.3% of patients with mild to moderate disease were vitamin D deficient. Mortality among patients with sufficient vitamin D was 2.3%, but 25.6% in vitamin D deficient patients.

“This study contributes to a continually evolving body of evidence suggesting that a patient's history of vitamin D deficiency is a predictive factor associated with poorer COVID-19 clinical disease course and mortality,” said study co-author Michael Edelstein a professor at Bar-Ilan University. “It is still unclear why certain individuals suffer severe consequences of COVID-19 infection while others don’t. Our finding adds a new dimension to solving this puzzle.”

**Article 3 (Included in Study 1)**

Woodyatt, A. (2021, September 22). *People who eat more dairy fat have lower risk of heart disease, study suggests*. CNN.  <https://www.cnn.com/2021/09/22/health/dairy-cardiovascular-disease-intl-scli-wellness-scn/index.html#:~:text=People%20who%20eat%20more%20dairy,heart%20disease%2C%20study%20suggests%20%7C%20CNN>

**Eating Less Dairy Fat Increases Risk of Heart Disease, Study Suggests**

A lower consumption of dairy fat increases risk of cardiovascular disease (CVD) as compared to high intakes, according to new research studying some of the world’s biggest consumers of dairy products.

An international team of scientists studied the dairy fat consumption of 4,150 60-year-olds in Sweden – a country with one of the world’s highest levels of dairy production and consumption – by measuring blood levels of a particular fatty acid that is mostly found in dairy foods. Experts then followed the cohort for an average of 16 years to observe how many had heart attacks, strokes, and other serious circulatory events, and how many of them died.

After statistically adjusting for other known cardiovascular disease related factors including age, income, lifestyle, dietary habits and other diseases, researchers found that low levels of the fatty acid – indicative of a low intake of dairy fats – increased risk of cardiovascular disease.

The team then confirmed these findings in other populations after combining the Swedish results with 17 other studies involving a total of almost 43,000 people from the US, Denmark and the UK.

“While the findings may be partly influenced by factors other than dairy fat, our study does not suggest any harm of dairy fat per se,” Matti Marklund, senior researcher at the George Institute for Global Health in Sydney and joint senior author of the paper, said in a statement.

“We found that lower levels of the fatty acid increased risk of CVD. These relationships are highly interesting, but we need further studies to better understand the full health impact of dairy fats and dairy foods,” he said.

**Article 4 (Included in Study 1)**

Elliott, B. (2022, March 2). *Just 30-90 minutes of resistance training weekly decreases risk of premature death – new research.* The Conversation. [https://theconversation.com/just-30-90-minutes-of-resistance-training-weekly-decreases-risk-of-premature-death-new-research-178209](https://nam12.safelinks.protection.outlook.com/?url=https%3A%2F%2Ftheconversation.com%2Fjust-30-90-minutes-of-resistance-training-weekly-decreases-risk-of-premature-death-new-research-178209&data=05%7C02%7Cjinana%40clemson.edu%7Ce405a3419d964bf96d3f08dc4a0f4b8f%7C0c9bf8f6ccad4b87818d49026938aa97%7C0%7C0%7C638466676743615571%7CUnknown%7CTWFpbGZsb3d8eyJWIjoiMC4wLjAwMDAiLCJQIjoiV2luMzIiLCJBTiI6Ik1haWwiLCJXVCI6Mn0%3D%7C0%7C%7C%7C&sdata=PQ8ezgG6A8ecUDs1oslscZDBiL4IsUf5dcKT1y4WJ1g%3D&reserved=0)

**Just 30-90 minutes of resistance training weekly decreases risk of premature death – new research shows**

There’s long been evidence that moderate aerobic exercise (e.g., walking, running, or cycling) are good for your lifelong health and well-being. Research even shows us more active people also tend to live longer, healthier lives with lower rates of disease – including cancers, diabetes, and cardiovascular disease.

But what about resistance exercise – like lifting weights? While it’s thought these kinds of exercises are probably also good for health and longevity, less evidence has existed showing the benefits. But a recent study now shows that 30-90 minutes of resistance training a week may decrease risk of premature death from all causes by 10%-20%.

The team of researchers from three universities in Japan conducted a meta-analysis – meaning they pooled data from 16 separate studies looking at longevity, disease related factors, and resistance exercise. This allowed them to look at tens of thousands of participants altogether.

They found that 30-90 minutes of resistance exercise per week may strongly decrease risk of dying from all causes. More strikingly, they also found regularly performing more than three hours of strength training per week actually poses an increased risk of premature death.

They also found that the optimal amount of time spent resistance training varied when it came to preventing different diseases. For example, while 40-60 minutes of strength training per week may reduce the risk of cardiovascular disease. However, resistance training was shown to have no effect on the risk of some specific types of cancer, such as bowel, kidney or pancreatic.
